# Association of Birth Parameters with Refractive Status in a Sample of Caucasian Children Aged 4–17 Years

**DOI:** 10.1155/2015/635682

**Published:** 2015-04-09

**Authors:** Berna Akova-Budak, Sertaç Argun Kıvanç, Osman Okan Olcaysü

**Affiliations:** ^1^Department of Ophthalmology, School of Medicine, Uludag University, 16059 Bursa, Turkey; ^2^Department of Ophthalmology, Erzurum Region Education and Training Hospital, 25240 Erzurum, Turkey

## Abstract

*Purpose*. To investigate the association of birth parameters with refractive status in different age groups of Caucasian children. *Materials and Methods*. This cross-sectional study included 564 eyes of 282 children aged 4 to 17 years. All children underwent complete ophthalmologic examination. The children were divided into three groups according to their refractive status (emmetropia,myopia, and hyperopia), ages (4–7, 8-9, 10–12, and 13–17), and appropriateness for gestational age, respectively. *Results*. The mean age of the children was 9.2 ± 2.8 (age range 4–17 years). The mean spheric equivalent was +0.3 ± 1.7 (range: (−10.0)–(+10.0) diopters). The mean birth weight and gestational age were 2681.1 ± 930.8 grams (750–5000 grams) and 37.2 ± 3.7 weeks (25–42 weeks). According to multinominal logistic regression analysis, children with myopia were more likely to have higher birth weights than emmetropic children (OR: 1.0, 95% CI: 1.000–1.001, and *P* = 0.028). The hypermetropes were found to be significantly small for gestational age between 13 and 17 years of age. *Conclusion*. Birth weight and appropriateness for gestational age as birth parameters may have an impact on development of all types of refractive errors. The hypermetropic children tended to be small for gestational age.

## 1. Introduction

Full term newborn babies tend to be hypermetropic at birth [1, 2] whereas preterm babies have been found to be either hypermetropic or myopic; even very preterm babies with severe ROP are slightly hyperopic at birth [3–5].

To date, refractive status at birth and its relation to birth weight (BW), birth length, head circumference, and gestational age (GA) have been studied and refractive error has been reported to correlate better with birth weight more than it did with GA [6]. On the one hand, another study on preterms from 2 weeks to 6 months of age reported no correlation of refractive error to GA or BW [7].

Babies born prematurely, with low birth weight or with retinopathy of prematurity, usually develop myopia [3, 8]. Numerous studies have been carried out to find out the relations of refractive error and ocular biometric measures with birth parameters in preterm or low birth weight child at birth and in the long term [6, 9–13]. A few studies investigated the association of birth parameters and refraction or biometric parameters in the general population of children [14–16]. In this cohort study, we aimed to investigate the association of birth parameters (BW, GA, and appropriateness for GA) with refractive status in different age groups of Caucasian children.

## 2. Materials and Methods

This cross-sectional study included children aged 4 to 17 years who had been examined at the Department of Ophthalmology in 2013. The children with neurologic sequela such as mental retardation and cerebral palsy and those who had ocular diseases such as glaucoma, cataract, and history of treatment for retinopathy of prematurity and those who had undergone any ocular surgery were excluded. In addition, the children with missing data regarding birth parameters were also excluded. A total of 282 children were enrolled in the study. Verbal assent was obtained from all children. Written informed consent was taken from the parents of the children who were involved in the study. The study was in accordance with the standards of the local ethics committee and conformed to the tenets of the Declaration of Helsinki.

The parents of the children provided the information regarding BW and GA based on the child's hospital delivery record.

All children underwent complete ophthalmologic examination including measurement of Snellen visual acuity as decimals, refraction, and dilated funduscopy. For cycloplegia, cyclopentolate 0.85% and phenylephrine 1.5% were instilled. After 30 minutes, the cycloplegic refraction measurement was performed with an autorefractor (Topcon A6300, Topcon Corporation, Tokyo, Japan). A total of 3 measurements were taken for each eye. The average value was recorded and the refraction was expressed as spherical equivalent (SE). The SE is the sum of spherical value and half of the cylindrical value. The children were divided into three groups according to their refractive status: emmetropia, myopia, and hyperopia. Emmetropia was defined between −0.5 and +0.5 D, myopia as less than −0.5, and hyperopia as more than +0.5, respectively. They were further divided into age groups: 4–7, 8-9, 10–12, and >13 years. Birth parameters as BW and GA were evaluated for each age group. They were also divided into small for gestational age (SGA), appropriate for gestational age (AGA), and large for gestational age (LGA) according to their birth weights for the gestational age. The international standardized average fetal growth tables were considered to calculate appropriateness for gestational age [17].

### 2.1. Statistical Analysis

For statistical analysis, SPSS 22 statistical program was used. Multinominal logistic regression analysis was used to evaluate the effect of BW, GA, and age at examination on refractive error. Pearson chi-square test was performed to compare qualitative data. The statistical significance was set at *P* < 0.01 and *P* < 0.05.

## 3. Results

The mean age of the children was 9.2 ± 2.8 (age range 4–17 years). Of 282 children, 155 were male (55%) and 127 were female (45%). The mean SE was +0.3 ± 1.7 (range: (−10.0)–(+10.0) diopters) and the mean Snellen visual acuity was 0.9 ± 0.1 (0.2–1.0). The mean BW and GA were 2681.1 ± 930.8 grams (750–5000 grams) and 37.2 ± 3.7 weeks (25–42 weeks). The mean age, BW, and GA distribution according to age groups and refractive status are given in Table 1. According to multinominal logistic regression analysis, children with myopia were more likely to have higher BWs than emmetropic children (OR: 1.0, 95% CI: 1.000–1.001, and *P* = 0.028). Myopia is also more likely to be found in older age groups than emmetropia (OR: 1.119, 95% CI: 1.27–1.219, and *P* = 0.010) and hypermetropia (OR: 1.39, 95% CI: 1.236–1.565, and *P* < 0.0001). Number of AGA, SGA, and LGA children and number of children under and over 2500 grams, according to refractive states in each age group, are given in Table 2. Emmetropic children aged between 4–7 and 8-9 years of age had significantly lower BWs under 2500 grams whereas myopic children aged between 4 and 7 years of age had significantly higher BWs over 2500 grams. The hypermetropes were found to be significantly SGA between 13 and 17 years of age.

## 4. Discussion

Birth weight, as one of the indicators of intrauterine development, may be associated with several systemic disorders in the long term [18]. Since it also affects the eye size, it may have an impact on refractive status or development of refractive error along with gestational age. Likewise, another parameter, appropriateness for gestational age, as SGA, AGA, and LGA may have an impact on refraction. Therefore, in our study, we also grouped the children as SGA, AGA, or LGA.

We found that children with myopia were more likely to have higher BWs than emmetropic children. Also, when the cut-off for birth weight is chosen as 2500 grams, the myopes had significantly higher BWs above 2500 grams than the others in children aged ≤7. There are other studies that investigated the relation between birth parameters and refraction. A comprehensive study in 1413 Singapore Chinese children reported an association between birth size and ocular dimensions [14]. They did not show a relation between birth size and refraction. However, they included school children 7–9 years old only and fewer premature children. We had 282 children with a wider range of age. Another study on Australian children found that axial length and corneal radius were related to BW, birth length, and head circumference but showed no trend or associations for spheric equivalent refractions in relation to birth parameters [15]. Apart from our study, they analysed children aged 6 years, in whom myopia can still develop, and excluded children who had been born before 36 weeks of gestational age. Similarly, a recent study also has reported that refraction is unrelated to birth size or gestational age in Swedish children aged 4–15 years [16], but this study comprised 143 children and did not include children born at <35 weeks' gestation.

Grjibovski et al. reported a significant association of BW with myopia in discordant dizygotic twin pairs [19]. On the other hand, another twin study with an age range of 18–86 years showed no association of BW with myopia [20]. But both studies showed that BW was not a major contributor to the discordance in myopia for monozygotic twin pairs. A cohort twin study comprising 1498 twins aged 5–80 years found no association between BW and refraction status. Lower BW tended to have shorter axial length and steeper corneas [21]. In our study, hypermetropia was more common in SGA children than in AGA or LGA children aged 13 years and older. These results may suggest that emmetropic SGA children in early ages tended to be hypermetropes in later ages. Our finding of increased hypermetropia in SGA is in accordance with the study of Lindqvist et al. [10]. They studied adolescents with very low birth weight (<1500 g), adolescents born at term but SGA, and concluded that being SGA may be a risk factor for hypermetropia development in adolescence, probably due to smaller eye size and other development arresting factors suggesting restricted fetal growth.

Prematurity has been associated with increased refractive errors including mainly myopia [22, 23]. However, it is also associated with hypermetropia [24]. Verma et al. also reported an inverse relationship between gestational age and incidence of refractive error [25]. In a cohort study comprising low birth weight children who were born preterm, the prevalence of all refractive errors was reported to be higher than the control group born at term who were involved in another study [13, 23]. In our study, we also included both premature and SGA children and found that hypermetropia is associated with being SGA. Myopic children had higher BWs than emmetropes. The discrepancy between our study and the others may result from other factors that have a role in inducing myopia. Myopia has been shown to be affected by environmental factors, such as near work, educational access, and urbanization as well as genetic factors [26, 27].

To sum up, birth weight and appropriateness as birth parameters may have an impact on development of all types of refractive errors. Being SGA may be associated with hypermetropia. Therefore, delivery records of children as birth weight and gestational age may be important in determining the follow-up of them for refractive state changes.

## Figures and Tables

**Table 1:** The mean age, birth weight, and gestational age distribution according to age groups and refractive status.

		Age			Birth weight (gram)		Gestational age (week)	
		*N*	Mean	SD	Mean	SD	Mean	SD
Age groups (Patients)	≤7	106	6,29	,806	2422,36	1019,306	35,58	4,431
	8-9	53	8,44	,513	2624,34	838,278	37,25	3,524
	10–12	77	10,91	,843	2865,58	782,759	38,44	2,208
	≥13	46	13,67	1,223	3033,91	875,713	38,61	2,349
	Total	282	9,16	2,846	2681,10	930,792	37,17	3,690

Refractive status (Eyes)	Myopia	91	10,38	2,653	3019,89	799,106	38,23	2,617
	Emmetropia	372	9,20	2,849	2601,42	935,861	36,92	3,895
	Hypermetropia	101	7,92	2,493	2669,31	961,987	37,15	3,601
	Total	564	9,16	2,846	2681,10	930,792	37,17	3,690

*N*: number; SD: standard deviation.

**Table 2:** Number of eyes in AGA, SGA, and LGA groups and in children under and over 2500 grams, according to refractive states in each age group.

Age groups (year)	Refractive status	Total *N*	AGA			SGA			LGA			≤2500 gr			>2500 gr		
			*N*	% in row	% in column	*N*	% in row	% in column	*N*	% in row	% in column	*N*	% in row	% in column	*N*	% in row	% in column
≤7	Myopia	18	12	66	10	4	22	6	2	12	7	6	33	5	**12**	**67** ^p^	**13**
	Emmetropia	134	78	58	66	41	31	60	15	11	58	**86**	**64**	**72** ^p^	48	36	52
	Hypermetropia	60	28	46	24	23	38	34	9	16	35	28	47	23	32	53	35
	Total	212	118	N/A	100	68	N/A	100	26	N/A	100	120	N/A	100	92	NA	100

8-9	Myopia	15	13	87	18	2	13	6	0	N/A	N/A	3	20	7	12	80	19
	Emmetropia	79	48	61	67	**31**	**39**	**91** ^p^	0	N/A	N/A	**38**	**48**	**86** ^p^	41	52	66
	Hypermetropia	12	11	92	15	1	8	3	0	N/A	N/A	3	25	7	9	75	15
	Total	106	72	N/A	100	34	N/A	100	0	N/A	N/A	44	N/A	100	62	N/A	100

10–12	Myopia	39	15	38	21	21	54	30	3	8	25	17	44	27	22	56	24
	Emmetropia	93	46	44	64	38	42	54	9	14	75	34	37	55	59	63	64
	Hypermetropia	22	11	50	15	11	50	16	0	0	0	11	50	18	11	50	12
	Total	154	72	N/A	100	70	N/A	100	12	N/A	100	62	N/A	100	92	N/A	100

≥13	Myopia	19	14	74	24	3	16	10	2	10	50	4	21	15	15	79	23
	Emmetropia	66	43	65	74	21	32	70	2	3	50	17	26	65	49	74	74
	Hypermetropia	7	1	14	2	**6**	**86** ^*^	**20**	0	0	0	**5**	**71** ^p^	**20**	2	29	3
	Total	92	58	N/A	100	30	N/A	100	4	N/A	100	26	N/A	100	66	N/A	100

^*^Fisher's exact test.

^p^Pearson chi-square test.
